# Computer-Aided Drug Design Across Breast Cancer Subtypes: Methods, Applications and Translational Outlook

**DOI:** 10.3390/ijms262110744

**Published:** 2025-11-05

**Authors:** Wei Tian, Ying Hu, Xinyu Gao, Jinghui Yang, Wei Jiang

**Affiliations:** School of Life Science and Technology, Wuhan Polytechnic University, Wuhan 430023, China

**Keywords:** Computer-aided drug design, luminal, HER2-positive, triple-negative breast cancer, artificial intelligence, machine learning

## Abstract

Breast cancer is a heterogeneous malignancy with distinct molecular subtypes that complicate the development of effective therapies. Traditional drug discovery methods are often constrained by high cost and long development timelines, underscoring the need for more efficient, subtype-aware approaches. Computer-aided drug design (CADD) has emerged as a valuable strategy to accelerate therapeutic discovery and improve lead optimization. This review synthesizes advances from a subtype-centric perspective and outlines the application of CADD techniques, including molecular docking, virtual screening (VS), pharmacophore modeling, and molecular dynamics (MD) simulations, to identify potential targets and inhibitors in receptor-positive (Luminal), HER2-positive (HER2^+^), and triple-negative breast cancer (TNBC). In addition to traditional pipelines, we highlight artificial intelligence (AI)-enabled methods and a hybrid workflow in which learning-based models rapidly triage chemical space while physics-based simulations provide mechanistic validation. These approaches have facilitated the discovery of subtype-specific compounds and enabled the refinement of candidate drugs to enhance efficacy and reduce toxicity. Despite these advances, critical challenges remain, particularly tumor heterogeneity, drug resistance, and the need to rigorously validate computational predictions through experimental studies. Future progress is expected to be driven by the integration of AI, machine learning (ML), multi-omics data, and digital pathology, which may enable the design of more precise, subtype-informed, and personalized therapeutic strategies for breast cancer.

## 1. Introduction

Breast cancer is the most commonly diagnosed malignancy among women worldwide and still represents a leading cause of cancer-related mortality despite major advances in screening and systemic therapies [1,2]. Its clinical management is strongly influenced by molecular heterogeneity, with major subtypes including hormone Luminal, HER2-positive (HER2+), and triple-negative breast cancer (TNBC), each showing distinct therapeutic vulnerabilities [3]. Although targeted therapies and endocrine interventions have improved outcomes in selected groups, intrinsic and acquired resistance continue to limit long-term benefits, underscoring the need for novel treatment strategies [4,5,6]. Across subtypes, recurrent challenges include endocrine resistance associated with estrogen receptor 1 (ESR1) mutations in luminal disease, pathway rewiring (e.g., RTK-PI3K-AKT-mTOR) and central nervous system involvement in HER2^+^ disease, and genomic instability with target scarcity and immune heterogeneity in TNBC [4,5,6]. This heterogeneity highlights the importance of computational approaches tailored to subtype-specific vulnerabilities. At a finer granularity, luminal disease subdivides into luminal A (ER/PR positive, lower proliferation, better prognosis) and luminal B (ER/PR positive with higher Ki-67 and/or HER2 co-expression, more aggressive); HER2^+^ tumors are driven by ERBB2 amplification or overexpression, and TNBC (ER/PR/HER2 negative) often shows basal-like, aggressive biology [1,2]. Treatment aligns with subtype (endocrine therapy with or without CDK4/6 inhibitors for luminal; HER2-directed antibodies, antibody-drug conjugates, or tyrosine kinase inhibitors (TKIs) for HER2^+^ [3,5]; chemotherapy with or without immunotherapy for TNBC [4]), but resistance remains common ESR1 mutations and ER rewiring in luminal [6]; pathway reactivation and brain metastases in HER2^+^ [3]; scarce targets and immune evasion in TNBC [4].

Computer-aided drug design (CADD) has become a central paradigm in oncology research, leveraging computational modeling to accelerate the identification, optimization, and personalization of anticancer therapeutics [7,8]. Advances in molecular docking, molecular dynamics (MD) simulations, relative binding free-energy (RBFE) calculations, and machine learning (ML) have expanded CADD from predicting ligand-receptor binding to supporting multi-omics-driven systems pharmacology [9,10,11]. The integration of AI further enables accurate prediction of drug-target interactions, absorption, distribution, metabolism, excretion, and toxicity (ADMET) properties, and resistance mechanisms, thereby facilitating the rational design of modalities such as selective estrogen receptor degraders (SERDs), HER2-targeting antibodies, and Proteolysis-Targeting Chimeras (PROTACs) [12,13,14]. In practice, these methods link structural and dynamic models with data-driven analytics to generate decision-grade, subtype-aware hypotheses that can be prospectively tested [9,10,11].

Recent applications highlight the versatility of CADD across breast cancer subtypes. In luminal disease, structure-guided optimization has accelerated the development of next-generation oral SERDs, such as elacestrant and camizestrant, which have demonstrated clinical benefit in patients with ESR1-mutant advanced breast cancer [15,16,17,18,19]. In HER2^+^, computational design has guided antibody engineering, kinase inhibitor optimization, and PROTAC development, generating promising options against resistant tumors [20,21]. For TNBC, where therapeutic targets remain limited, CADD has contributed to exploiting DNA repair deficiencies, developing poly(ADP-ribose) polymerase (PARP) inhibitors, and supporting precision immunotherapy through AI-based biomarker discovery [22,23,24]. Beyond these examples, structure- and AI-guided design [10] is increasingly used to inform antibody-drug conjugate payload and linker selection, optimize ternary-complex geometry for HER2^−^ [20] or ER^−^ targeting PROTACs, and identify epigenetic modulators or dual-target scaffolds for TNBC [14], supporting rational combinations with chemotherapy and immunotherapy [22].

Despite substantial progress, several critical challenges remain. These include incomplete structural coverage, heterogeneous validation, limited availability of high-quality structural data for membrane proteins and multi-subunit complexes, imperfect toxicity and multi-target modeling, and dataset biases that constrain generalizability [25,26,27]. In this review, we outline the structural foundations and end-to-end workflow of CADD, examine subtype-specific applications in luminal, HER2^+^, and TNBC, and summarize enabling methodologies such as docking, MD with RBFE calculations, quantitative structure-activity relationship (QSAR), ADMET modeling, and generative AI, along with their current limitations. We also contrast traditional and AI-enabled workflows to clarify how learning-based modules can complement physics-based refinement within subtype-aware pipelines. Finally, we discuss translational considerations, ranging from biomarker selection to rational therapeutic combinations. Through this integration, more effective and subtype-specific therapies are expected to benefit patients across diverse forms of breast cancer.

## 2. Foundations and Workflow of Computer-Aided Drug Design (CADD) in Cancer Therapeutics

### 2.1. Structural Foundations and Computational Methods

**Figure 1:**
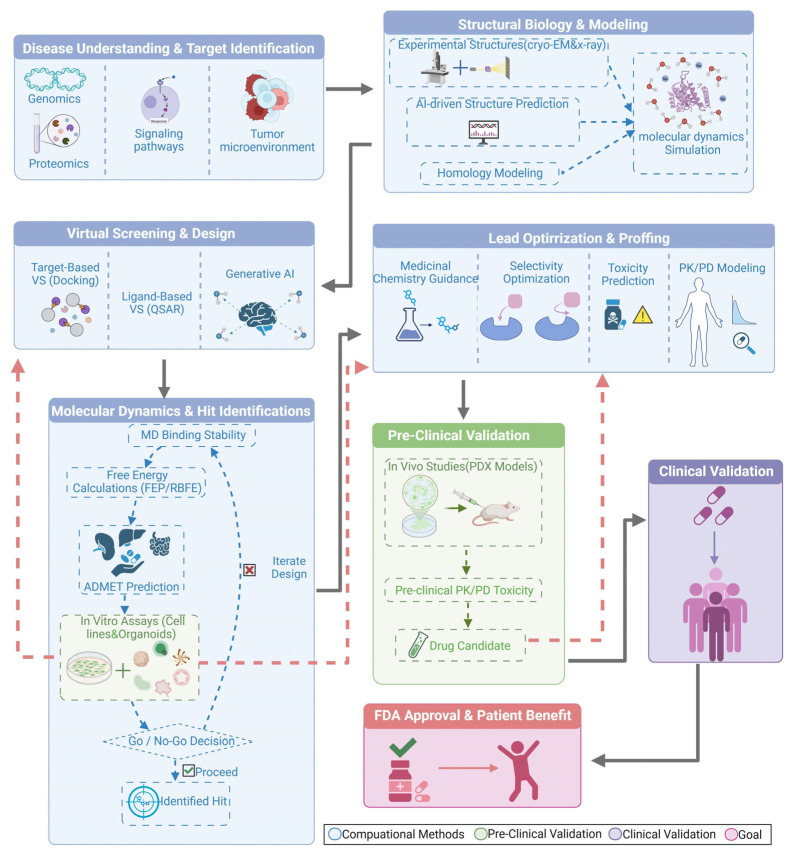
End-to-end CADD workflow for cancer therapeutics. The schematic illustrates an integrated CADD pipeline that links disease understanding, target identification, and structural biology with experimental structures (cryo-EM, X-ray crystallography), AI-based structure prediction, and molecular dynamics (MD) modeling. Virtual screening (VS) and design incorporate target-based docking, quantitative structure-activity relationship (QSAR) modeling, and generative AI. Lead optimization includes medicinal chemistry, selectivity optimization, toxicity prediction, and pharmacokinetics (PK)/pharmacodynamics (PD) modeling. MD-based hit identification evaluates relative binding free-energy (RBFE) calculations, and absorption, distribution, metabolism, excretion, and toxicity (ADMET) properties. Preclinical validation, including patient-derived xenograft (PDX) studies, supports progression to clinical validation and FDA approval. Color key: blue, computational methods; green, preclinical validation; purple, clinical validation; pink, goal. Created in BioRender. Yang, J. (2025) https://BioRender.com/zvc3fmc.

CADD critically depends on accurate three-dimensional representations of molecular targets. The overall workflow is summarized in Figure 1. When experimental coordinates are unavailable or incomplete, homology modeling and MD are used to refine binding-site geometries and explore relevant conformational ensembles. High-accuracy predictors such as AlphaFold 3 and ColabFold routinely provide starting models that can be refined and validated with MD before iterative design [28,29,30,31]. For protein assemblies, AlphaFold-Multimer offers useful predictions but has clear limitations in multi-chain complexes, which often require complementary experimental data or restrained MD refinement [32]. When experimental structures are unavailable, template-based homology modeling (e.g., SWISS-MODEL, MODELLER, RosettaCM) and threading provide decision-grade starting models. Recommended practice includes template quality assessment, loop remodeling, restrained MD relaxation, and orthogonal validation (e.g., mutational constraints) prior to docking/RBFE. These approaches complement AI predictors and often seed production-level CADD campaigns [33,34,35,36,37,38].

The growing interest in targeted protein degradation underscores the need for structural precision. PROTAC-based strategies depend on productive ternary-complex formation, so contemporary workflows integrate linker conformational sampling, analysis of protein-protein interfaces, and treatment of cooperative binding energetics. Docking and MD, often combined with enhanced sampling, are used to prioritize chemotypes that stabilize the ternary complex [33]. Predicted structures of both the protein of interest and the E3 ligase frequently seed these studies, and cryo-EM or crystallography provides orthogonal confirmation before lead optimization [34].

Structure-based VS employs classical docking to enumerate poses and estimate affinities, with AutoDock family programs and commercial engines remaining standard for large-scale library exploration [35]. Learning-based pose generators, such as DiffDock and EquiBind, accelerate conformational sampling and enable hybrid pipelines in which deep-learning outputs are subsequently rescored using physics-based methods [36,37]. Ligand-based modeling also remains essential for identifying structure-activity trends; modern deep QSAR, trained on curated datasets, improves predictive accuracy and guides multi-parameter optimization, including ADMET and developability considerations [38]. Furthermore, for potency refinement, RBFE calculations based on alchemical methods and λ-dynamics provide quantitative ΔΔG estimates when rigorous system preparation and sampling protocols are enforced. At the initial stage of the pipeline, generative design frameworks, including diffusion models, language models, and reinforcement learning, propose synthetically accessible chemotypes aligned with pharmacological and ADMET requirements, feeding candidates back into the screening-and-refinement loop [39]. The overall CADD-assisted drug-development process is summarized in Figure 1.

### 2.2. Applications of CADD Across Breast Cancer Subtypes

The clinical and molecular heterogeneity of breast cancer necessitates subtype-specific design strategies, and CADD has emerged as a versatile tool to support such tailored interventions. In luminal subtypes, computational workflows have facilitated the development of next-generation SERDs and related molecules that overcome endocrine resistance by accounting for receptor pocket plasticity and mutational landscapes within docking, QSAR, and RBFE pipelines [40]. In HER2-positive disease, structure prediction and antibody/kinase-inhibitor modeling inform affinity maturation and selectivity optimization, while physics-based rescoring helps discriminate among compounds with subtle hinge-binding or allosteric differences [41]. For TNBC, multi-omics-guided target triage integrated with structure- and ligand-based prioritization has advanced PARP-centered therapies and epigenetic modulators, with AI-driven models further supporting biomarker discovery and drug sensitivity prediction [42,43].

Across all subtypes, predicted structures have become integral to CADD pipelines when experimental models are unavailable. Best practices emphasize confidence annotation, targeted relaxation, and hybrid workflows that integrate prediction with experimental validation, thereby ensuring decision-quality hypotheses [28,29,30,31,32,34].

## 3. CADD Strategies for Luminal Breast Cancer

### 3.1. Targeting Estrogen Receptor Degradation

**Figure 2:**
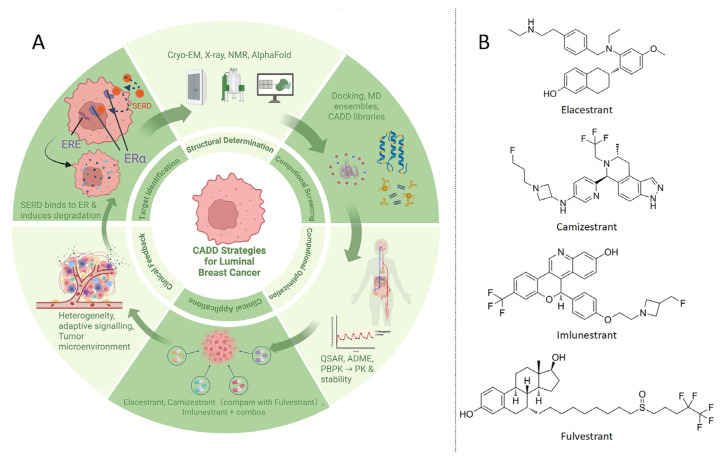
CADD strategies for luminal breast cancer and representative agents. (**A**) An iterative CADD workflow for ESR1-mutant luminal breast cancer is delineated. SERDs are employed to lower ER levels and suppress signaling. Structural determination by cryo-electron microscopy cryo-EM is linked to VS through docking, QSAR modeling, and MD simulations. Lead optimization is undertaken with medicinal chemistry guidance, selectivity profiling, absorption, distribution, metabolism, and excretion ADME assessment, and PK/PD modeling, while in vitro and preclinical studies support go/no-go decision making and iteration. (**B**) Representative SERDs structures: elacestrant, camizestrant, fulvestrant and imlunestrant. Created in BioRender. Yang, J. (2025) https://BioRender.com/2yq423v.

A central objective of CADD in luminal breast cancer is the development of SERDs. Unlike conventional endocrine therapies that competitively block estrogen binding, SERDs promote receptor degradation, thereby reducing ER levels and silencing downstream signaling. This mechanism is particularly effective against ESR1-mutant tumors, which frequently develop resistance to first-line therapies such as tamoxifen. In parallel with these advances, it is worth noting that fulvestrant, approved by the FDA in 2002, was the first SERD to enter clinical use, underscoring the historical reliance on injectable agents and the unmet need that has driven the development of optimized oral degraders [44,45,46,47]. An overview of the iterative CADD workflow is provided in Figure 2A.

CADD methodologies, including structure-based drug design, VS, and MD simulations, have been instrumental in identifying small molecules capable of binding the ER ligand-binding domain (LBD) and triggering degradation. Elacestrant, the first oral SERD approved for advanced ER-positive breast cancer, exemplifies this strategy by demonstrating robust activity in clinical trials and efficacy against ESR1 mutations [48,49,50,51,52]. Similarly, GDC-0810, discovered and optimized through CADD, showed preclinical efficacy and advanced into clinical testing as a promising degrader [53]. Next-generation oral SERDs such as camizestrant and imlunestrant have also demonstrated encouraging results in recent clinical trials [54,55,56]. Beyond these, newer investigational agents such as giredestrant have also shown meaningful activity, reinforcing the therapeutic value of ER-targeted degradation strategies.

Several pivotal clinical trials have further highlighted the progress of SERD development. The SERENA-2 trial (phase II, camizestrant in ER^+^/HER2^−^ advanced breast cancer) demonstrated that camizestrant significantly prolonged progression-free survival (PFS) compared with fulvestrant in ESR1-mutant patients [54]. The EMBER trial (phase II, imlunestrant) defined the phase 2 dose of imlunestrant with favorable safety and efficacy [55]. Furthermore, abemaciclib-based combination regimens achieved superior outcomes in endocrine-resistant settings [56], while updated subgroup analyses from the EMERALD trial (phase III, elacestrant) reaffirmed the clinical benefit of elacestrant in ESR1-mutant populations [47,49]. Collectively, these developments highlight the continuum from injectable SERDs to optimized oral degraders, with clinical benefits increasingly evident across ESR1-mutant subgroups. These advances also underscore the need for deeper mechanistic understanding, where structural biology provides critical insights to guide the rational design of next-generation degraders. Representative SERD structures are shown in Figure 2B.

### 3.2. Structural Insights and Advanced Design Strategies

Building on the clinical advances achieved with SERDs, structural biology has provided critical insights that inform the next generation of degrader design. High-resolution techniques such as cryo-EM, X-ray crystallography, and NMR have enabled precise characterization of estrogen receptor conformations [52,57]. These structural data support rational drug design by revealing novel binding pockets and guiding the optimization of ligand-receptor interactions. Mechanistically, ligand-induced ER conformational changes dictate AF-domain engagement and co-regulator recruitment, providing a structural rationale for how degraders and antagonists remodel transcriptional output.

Recent applications of cryo-EM have clarified the ER-LBD complex of GDC-0810 at atomic resolution, directly guiding chemical modifications that improved binding specificity [53,57]. Complementary approaches such as X-ray crystallography and NMR spectroscopy have offered additional high-resolution views of ligand-receptor interactions, revealing conformational rearrangements that dictate binding and degradation efficiency. Computational tools including AlphaFold have further expanded this structural landscape by predicting ESR1 mutant conformations and uncovering previously inaccessible druggable pockets [58,59]. When integrated with MD simulations, these models capture conformational ensembles and expose transient grooves and allosteric sites that can be exploited for novel ligand design. Structural mapping of such allosteric sites has already informed concepts for next-generation SERDs that combine orthosteric antagonism with allosteric modulation [52]. By coupling these diverse structural insights with CADD-driven VS, docking, and free-energy calculations, large chemical libraries can be interrogated with higher precision. This hybrid approach not only accelerates the discovery of SERDs with improved potency and selectivity but also provides a framework for anticipating resistance-associated mutations and rationally designing compounds that retain activity in mutant contexts [54,60]. The end-to-end CADD process, integrating structural determination, computational screening, lead optimization, and clinical feedback, is summarized in Figure 2A.

Importantly, recent computational advances have revealed unique conformations of ESR1-Y537S and D538G mutants. AI-based structural modeling, including AlphaFold 3 and MD simulations, has enabled the design of ligands with improved mutant selectivity. In parallel, structural studies have mapped allosteric sites, paving the way for next-generation SERDs with dual mechanisms of action [52]. These structural principles also help explain the tissue-selective outcomes historically seen with SERMs such as raloxifene, motivating designs that bias AF-domain states toward degradation or deep antagonism.

### 3.3. Overcoming Pharmacokinetic Challenges Through CADD

Beyond target engagement, the clinical performance of SERDs is critically determined by their PK properties, including ADME. Efficacy can be severely limited when poor oral bioavailability or rapid clearance is observed. The importance of early in-silico ADME screening is underscored by lessons learned from classic endocrine therapies: for example, tamoxifen is metabolized primarily to *N*-desmethyltamoxifen (~92%) with only a minor 4-hydroxytamoxifen branch (~7%), and the activity of its metabolites is further influenced by efflux through ABC transporters. Such liabilities, if anticipated computationally, can be mitigated to shorten optimization cycles for oral SERDs. In this context, in-silico approaches provided by CADD, including QSAR modeling and ADME prediction, are increasingly applied to enable early optimization of PK profiles [59,60,61,62].

For instance, elacestrant underwent iterative optimization guided by QSAR and ADME modeling, leading to improved metabolic stability, oral exposure, and favorable PK characteristics [48,49,50,51,52]. Similarly, AZD9496 followed a CADD-driven optimization process in which scaffold modifications reduced oxidative metabolism and improved bioavailability, ultimately yielding a clinically favorable PK profile [60,61,62,63,64]. Analogously, prospective flags for CYP-mediated clearance and transporter interactions are now being built into early triage criteria, streamlining progression to candidates with stable oral exposure.

Collectively, these examples illustrate how CADD-based PK/ADME optimization can rationally address metabolic instability and bioavailability challenges, thereby accelerating the progression of oral SERDs into clinical development. Together with emerging computational-experimental frameworks, such as physiologically based pharmacokinetic (PBPK) simulations integrated with ML, these advances underscore the potential of CADD to overcome pharmacokinetic barriers and facilitate clinical translation. In line with this, recent reviews have highlighted that oral SERDs developed between 2023 and 2025 exhibit improved half-lives and reduced CYP450-mediated clearance through scaffold modifications guided by computational prediction [60,61,62]. Moreover, integrated models combining PBPK simulations with ML are now being applied to personalize SERD dosing strategies, further extending the scope of CADD in pharmacokinetic optimization [61].

### 3.4. Clinical Applications and Translational Challenges of CADD-Designed SERDs

Despite substantial progress, the clinical translation of CADD-designed SERDs continues to face important obstacles. Tumor heterogeneity, the complexity of the tumor microenvironment, and the evolution of resistance mechanisms limit the durability and universality of therapeutic benefit [64]. To illustrate how CADD contributes across the developmental continuum of SERDs in luminal breast cancer. Clinically, palbociclib, a CADD-optimized CDK4/6 inhibitor, has demonstrated substantial benefit when combined with letrozole in ER-positive breast cancer [65,66]. Building on this paradigm, elacestrant is being evaluated in combination with CDK4/6 and PI3K/AKT/mTOR inhibitors, underscoring the role of CADD in rationally designing combination regimens [48,50]. The SERENA-6 trial further showed that ctDNA-guided early switching to camizestrant plus CDK4/6 inhibition significantly prolonged PFS compared with continued aromatase inhibitor therapy [67]. Real-world evidence has also validated the clinical utility of routine ESR1 mutation testing, providing a practical framework for selecting patients most likely to benefit from SERD-based regimens [68]. Beyond ER targeting, the androgen receptor (AR) axis is under active investigation, with selective AR modulators such as enobosarm demonstrating activity in ER-positive settings, thereby offering a complementary endocrine strategy.

Nevertheless, key challenges remain. Tumor heterogeneity, adaptive signaling, and microenvironmental influences continue to drive therapeutic resistance, emphasizing the need for refined CADD strategies that integrate multi-omics data, model dynamic resistance pathways, and inform rational design of combination therapies [54,55,56,67,69,70]. Furthermore, the relative balance of ERα and ERβ isoforms has been implicated in endocrine sensitivity and resistance, suggesting that biomarker-guided stratification could further optimize SERD-based regimens. Because resistance circuits frequently converge on RTK-PI3K-AKT-mTOR signaling and cell-cycle control, CADD-guided approaches are especially suited to the design of rational combination therapies. By integrating these strategies, SERD development has advanced from in silico discovery to clinical validation, though persistent challenges such as tumor heterogeneity and acquired resistance highlight the ongoing need for methodological innovation and rational therapeutic design.

## 4. CADD Strategies for HER2^+^ Breast Cancer

### 4.1. Targeting HER2 with Antibodies and Small Molecules

**Figure 3:**
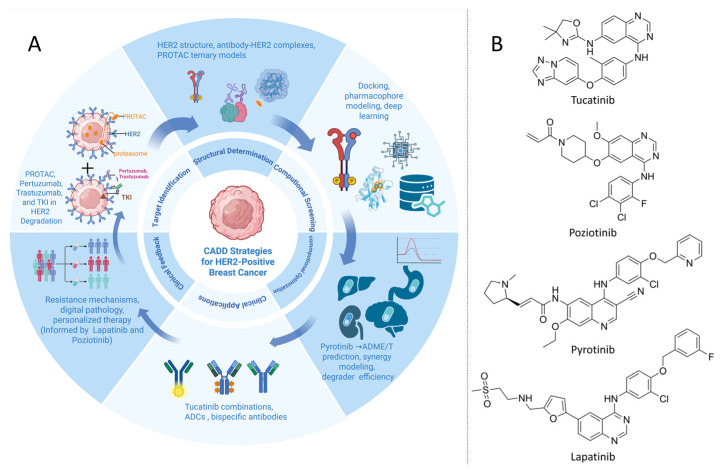
CADD strategies for HER2^+^ breast cancer and representative agents. (**A**) An iterative CADD workflow for HER2^+^ breast cancer is delineated. Structure-guided discovery and optimization are applied to HER2-directed modalities, including ADCs, PROTACs, and TKIs. Determination of HER2 structures, antibody HER2 complexes, and PROTAC ternary models is linked to virtual screening through docking, pharmacophore modeling, and deep learning, followed by MD simulations. Lead optimization is undertaken with medicinal chemistry guidance, selectivity profiling, ADMET prediction, and PK/PD modeling; degrader efficiency, resistance mechanisms, digital pathology, and patient stratification are incorporated to support go or no-go decision making and iterative design. (**B**) Representative TKIs structures: tucatinib, poziotinib, pyrotinib, and lapatinib. Created in BioRender. Yang, J. (2025) https://BioRender.com/9djmqa5.

The introduction of HER2-directed monoclonal antibodies, such as trastuzumab and pertuzumab, transformed the therapeutic landscape of HER2-positive breast cancer by blocking receptor activation and dimerization. Subsequently, antibody-drug conjugates (ADCs), including trastuzumab emtansine (T-DM1) and trastuzumab deruxtecan (T-DXd), further improved outcomes by coupling HER2 specificity with cytotoxic payloads. In parallel, CADD approaches such as docking, pharmacophore modeling, and MD simulations have been applied to optimize antibody binding, ADC linker stability, and small-molecule scaffolds, thereby enhancing affinity, selectivity, and therapeutic index [71]. An overview of CADD workflow and representative HER2-targeted agents is provided in Figure 3A.

TKIs represent a complementary therapeutic strategy. Early reversible inhibitors, such as lapatinib and neratinib, demonstrated clinical activity but were constrained by pharmacokinetic liabilities and toxicity. Structure-based design enabled the development of tucatinib, a highly selective HER2 TKI with proven clinical benefit, particularly in patients with brain metastases [72]. Innovations in antibody engineering, such as the bispecific antibody zanidatamab, further illustrate how computational modeling and AI-driven design can refine epitope recognition and anticipate resistance-associated mutations [73,74]. Nevertheless, therapeutic resistance frequently arises through receptor mutations, bypass signaling, or tumor heterogeneity, underscoring the need for novel strategies such as targeted protein degradation. Representative HER2 TKIs discussed here are shown in Figure 3B (tucatinib, poziotinib, pyrotinib, and lapatinib).

ADCs extend HER2 targeting by linking antigen recognition to payload delivery; performance hinges on antigen density/heterogeneity, internalization/trafficking, linker stability/cleavage, DAR, payload class, and bystander effects [43]. Reduced sensitivity arises from antigen loss or heterogeneity, trafficking changes, efflux, pathway rewiring, and high DNA-repair activity (for topo-I payloads), while CADD guides epitope selection, linker design, and payload choice through interface modeling, linker sampling, and risk assessment [28,34,43].

### 4.2. PROTACs for HER2 Degradation

Traditional HER2-targeted therapies primarily inhibit receptor activity, yet tumor cells often adapt through mutations or pathway reactivation, ultimately leading to therapeutic resistance. PROTACs introduce a distinct paradigm by harnessing the ubiquitin-proteasome system to eliminate HER2 rather than merely inhibiting its signaling. This strategy offers several potential advantages, including more durable pathway suppression, the ability to bypass resistance mutations that impair inhibitor binding, and reduced dosing requirements due to catalytic turnover of target degradation. Moreover, HER2 degraders may hold clinical value in patients with brain metastases, where conventional TKIs are often limited by poor blood–brain barrier penetration. Collectively, these features make PROTACs an attractive strategy for addressing the limitations of conventional HER2 blockade.

CADD has become integral to the rational design of HER2-targeted PROTACs. Key computational applications include optimizing linker length, flexibility, and geometry to promote productive ternary complex formation, as well as VS of warheads derived from clinically validated kinase scaffolds. docking enables simultaneous evaluation of interactions with HER2 and E3 ligases, while MD simulations provide insights into ternary complex stability, conformational adaptability, and ubiquitination likelihood. Recent studies underscore how CADD-guided design improves degradation efficiency, minimizes off-target liabilities, and informs the selection of appropriate ligases, such as cereblon or VHL, for HER2-specific degradation [75]. Design considerations for HER2 degraders align with the workflow summarized in Figure 3A.

Although HER2 PROTACs remain at the preclinical stage, emerging degraders have demonstrated robust activity in resistant cell lines and animal models, effectively suppressing downstream signaling and restoring therapeutic sensitivity. Computational modeling and AI-based prediction tools are streamlining the optimization of ternary complex stability and reducing potential off-target effects, particularly against receptors such as EGFR or HER3 [76]. While clinical translation will require validation of safety, pharmacokinetics, and selectivity, PROTACs highlight an important complementary strategy within the HER2 therapeutic landscape. By enabling targeted receptor elimination, they not only provide a conceptual framework for overcoming the limitations of inhibition-based therapies but also hold promise as a rational component of future combination regimens.

### 4.3. Combining CADD with Multi-Omics Data for Personalized Therapy

The heterogeneity of HER2^+^ breast cancer underscores the need for personalized therapeutic strategies. Integrating genomic, transcriptomic, proteomic, and pathology-derived data with CADD provides a comprehensive framework for precision therapy design [40,41,42]. By linking molecular alterations to structural and dynamic models, such integration also helps explain variable drug sensitivity and resistance patterns across patient subgroups [32,34]. Multi-omics-driven ML models trained on pre-treatment biopsies have identified tumor ecosystem features that correlate with therapeutic response, thereby offering valuable priors for target prioritization and regimen selection [77]. As summarized in Figure 3, this integrative approach forms a cyclical workflow that begins with target identification, proceeds through structural determination, VS, and lead optimization, and extends into clinical applications. Continuous clinical and multi-omics feedback then refines subsequent rounds of target discovery and drug optimization.

When integrated with multi-omics datasets, CADD enables modeling of drug-target interactions in the context of patient-specific molecular alterations. For example, TCGA-derived profiles combined with docking and MD simulations have shown that PI3K-mutant subgroups display reduced sensitivity to lapatinib, mainly due to activation of the PI3K-AKT-mTOR pathway and conformational alterations in the HER2 kinase domain that weaken inhibitor binding [77]. In addition, reduced sensitivity in HER2^+^ tumors may arise from HER3-mediated crosstalk with downstream MAPK signaling, increased drug efflux via ABC transporters, and mutations that remodel the ATP-binding site or the hinge-region hydrogen-bonding network [44,45,46,47,76,77]. Collectively, these alterations diminish target engagement and reactivate signaling despite continued drug exposure. CADD pipelines integrating structure-based modeling, MD simulations, and binding free-energy calculations can identify resistance-prone conformations and quantify their energetic penalties. These insights guide rational combinations of HER2 TKIs (lapatinib, tucatinib) with PI3K/AKT/mTOR inhibitors (alpelisib) or CDK4/6 inhibitors (palbociclib) to suppress compensatory signaling and restore therapeutic response [28,29,30,31,32,34,40,41,42,43]. By coupling structural insights with pathway-level modeling, CADD provides a mechanistic framework to explain and overcome reduced drug sensitivity across HER2^+^ subtypes.

Complementary evidence from patient-derived organoid studies indicates that tumors with high DNA repair pathway activity are more responsive to trastuzumab deruxtecan, further illustrating how structural modeling integrated with molecular profiling can guide rational drug selection [43,76]. Such approaches exemplify how computational pipelines can refine therapy design at the individual-patient level. AI-driven digital pathology and biomarker analyses further enhance these capabilities by enabling automated HER2-IHC assessment and predictive stratification tools. Deep learning-based models have been applied to evaluate HER2 expression heterogeneity from histopathology slides and correlate it with antibody binding predictions, thereby improving diagnostic reproducibility and forecasting therapeutic response [78,79]. Collectively, the integration of CADD with multi-omics and AI-based analytics exemplifies a precision oncology paradigm in which computational methods bridge molecular diversity with rational drug design.

### 4.4. Resistance Mechanisms and CADD-Guided Counterstrategies in HER2^+^ Breast Cancer

The strategies described in Section 4.1, Section 4.2 and Section 4.3 encompass established therapeutics such as antibodies, ADCs, and TKIs; emerging modalities such as PROTAC-mediated HER2 degradation; and precision frameworks integrating multi-omics and AI-based analyses. Collectively, they illustrate how computational approaches inform rational therapeutic design across multiple dimensions of HER2 targeting.

Despite these advances, resistance to HER2-targeted therapies remains a critical clinical challenge. Mechanisms include receptor mutations that impair antibody or TKI binding, activation of bypass signaling pathways such as PI3K/AKT and IGF1R, and protective influences from the tumor microenvironment. CADD provides systematic tools to model these resistance-associated alterations, predict their structural and functional impact, and guide the design of inhibitors or rational combinations capable of restoring therapeutic efficacy [80]. These mechanisms map onto the clinical-feedback node in Figure 3 and motivate rational combination strategies.

Mechanism-aware optimization can improve ADCs’ performance in genomically defined subgroups. Structural and trafficking models guide epitope selection to sustain internalization, while linker simulations and ADMET profiling help match cleavage motifs and payloads to tumor microenvironmental constraints [28,29,30,31,32,34,40,41,42,43]. When proteogenomic or digital-pathology data indicate HER2 heterogeneity, high efflux capacity, or enhanced DNA-repair activity, model-informed strategies include selecting more cleavable linkers, switching payload classes, or combining ADCs with HER2 TKIs or DDR inhibitors to restore sensitivity [43,76,80]. In parallel, variant-aware docking, MD, and RBFE analyses can prioritize TKIs against resistance-prone HER2 conformations and support network-guided combinations (e.g., HER2 TKI with PI3K/AKT or CDK4/6 inhibitors) to suppress compensatory signaling [28,29,30,31,32,34,40,41,42,43].

Clinically, tucatinib combined with trastuzumab and capecitabine has improved outcomes, including in patients with brain metastases, and updated analyses continue to refine its therapeutic role [72,81]. Moreover, the combination of tucatinib with T-DM1 has demonstrated prolonged PFS in a phase III trial, underscoring the translational value of rational combination design [82]. Beyond these, proteomics-informed systems approaches are identifying novel partners that counteract stromal protection and restore sensitivity to HER2-directed therapies [83]. Addressing these resistance mechanisms will be essential for extending the durability of HER2-targeted treatment and realizing the full potential of CADD-guided therapeutic innovation.

## 5. Multi-Dimensional CADD Strategies for Triple-Negative Breast Cancer (TNBC)

### 5.1. Computational Targeting of Molecular Pathways in TNBC

CADD workflows in TNBC often concentrate on signaling and repair networks that lack clinically validated targeted therapies. The PI3K/AKT/mTOR axis, DNA damage response (DDR), and epigenetic regulators remain high-value anchors for computational exploration. In particular, within the PI3K/AKT/mTOR pathway, first-line paclitaxel combined with AKT inhibitors demonstrated benefit in biomarker-enriched subgroups (e.g., PIK3CA/AKT1/PTEN alterations) in phase II trials. However, subsequent phase III trials, such as IPATunity130, failed to confirm this benefit in unselected TNBC populations, thereby highlighting a critical gap that can be addressed by CADD integrated with multi-omics to link mutational or phospho-proteomic profiles to drug responsiveness [84,85,86,87].

Moreover, in the DDR context, PARP inhibition remains clinically validated in TNBC, but resistance arises through BRCA1/2 reversion, homologous recombination (HR) restoration, PARG loss, and replication fork stabilization. CADD contributes by guiding the design of next-generation PARP inhibitors with optimized trapping profiles, modeling synergy with ATR/CHK1 or immune checkpoint partners, and proposing dual-warhead scaffolds. Rationally designed dual PARP/HDAC inhibitors have already demonstrated activity in TNBC cell models [88,89,90,91].

Additionally, epigenetic modulation represents another computationally tractable target space. BRD4, a central regulator of transcriptional programs in basal-like TNBC, has shown preferential pharmacologic vulnerability. Foundational studies mapped resistance circuitry, enabling scaffold optimization and rational combination strategies. CADD has supported BRD4-centered dual-target concepts (e.g., EGFR/BRD4 hybrids) that exhibit validated anti-proliferative activity in TNBC lines [92,93,94,95,96]. Importantly, BRD4 inhibition also downregulates PD-L1 expression, linking epigenetic blockade to checkpoint therapy design [97,98,99].

### 5.2. PROTACs Targeting Transcriptional Regulators in TNBC

Unlike HER2^+^ disease, where PROTACs mainly aim at receptor tyrosine kinases, TNBC PROTAC efforts focus on nuclear and transcriptional regulators. A BRD4-specific PROTAC was optimized through structural modeling and solvent mapping, demonstrating selective degradation via CRBN recruitment, suppression of basal-like TNBC growth, and disruption of the KLF5 super-enhancer program [100]. These degraders mechanistically align with the PD-L1/BRD4 axis, offering a rationale for immunotherapy combinations.

CADD-driven BRD4 PROTAC design emphasizes linker conformational sampling, E3 ligase positioning, and ternary complex dynamics, with pharmacokinetic simulations used to anticipate permeability and metabolic liabilities [101]. Although estrogen receptor-directed degraders are irrelevant in TNBC, lessons from BRD4-targeting PROTACs provide proof-of-concept. MD of ternary complexes suggests similar frameworks could be extended to other TNBC vulnerabilities, including NF-κB pathway components and DDR adaptors. Thus, CADD-guided degrader design in TNBC emphasizes transcriptional and chromatin regulators, distinguishing it from receptor-centric strategies in HER2^+^ disease.

### 5.3. Multi-Omics Integration for Personalized TNBC Therapy

The clinical heterogeneity of TNBC is recognized to surpass that of HER2-positive disease, encompassing basal-like, mesenchymal, immunomodulatory, and luminal-androgen receptor (LAR) subtypes. Target-scaffold pairs are increasingly prioritized through the integration of CADD with transcriptional subtyping [102,103,104]. For example, docking combined with subtype-specific gene expression signatures has been applied to rank BRD4 scaffolds for basal-like TNBC, whereas PI3K/AKT inhibitors have been considered more relevant in mesenchymal-enriched tumors. At the systems level, multi-omics ML predictors have been shown to reveal how pre-treatment tumor ecosystems influence therapeutic response and can be used to forecast pathological complete response or event-free survival. These predictors have also been employed as priors for in silico combination ranking and virtual patient enrichment [105,106,107,108].

In contrast to HER2^+^ disease, where pathology-derived biomarkers are predominantly used, TNBC has increasingly incorporated radiomics and radiogenomics. Imaging features integrated with computational pharmacology have been employed to predict therapeutic sensitivity and to inform dose scheduling tailored to phenotypic subgroups [109,110,111,112]. For instance, MRI-based radiogenomic signatures were evaluated in a cohort of 134 TNBC patients to predict pathological complete response to neoadjuvant chemotherapy, and a predictive performance with an area under the curve (AUC) close to 0.80 was reported, thereby illustrating how imaging data can be leveraged to guide therapy design [111]. Taken together, while HER2-positive CADD frameworks are mainly directed toward refining established antibody and TKI therapies, TNBC strategies are being advanced through the use of transcriptional subtypes, tumor ecosystem features, and imaging-derived markers to inform novel drug design and patient selection.

### 5.4. Resistance Mechanisms and CADD Counterstrategies in TNBC

The CADD-driven strategies described in Section 5.1, Section 5.2 and Section 5.3 encompass pathway-focused targeting of PI3K/AKT, DDR, and epigenetic regulators; emerging PROTAC applications against transcriptional drivers such as BRD4; and integrative multi-omics approaches for personalized therapy. Collectively, they highlight how computational approaches can inform both molecular design and patient stratification in TNBC.

Despite these advances, TNBC remains highly prone to therapeutic resistance. Mechanisms include pathway rewiring, drug efflux pumps, tumor persister states, and restoration of DNA repair capacity. CADD contributes by modeling liabilities such as P-glycoprotein recognition, simulating synergistic combinations (e.g., PARP+ATR/CHK1, PARP+PD-(L)1), and nominating ADC or degrader partners to reduce single-target escape [113]. Clinically, combinations such as pembrolizumab with chemotherapy or sacituzumab govitecan with pembrolizumab illustrate how rational design can be aligned with immunotherapy [114,115,116,117,118]. For PARP inhibitor resistance, mechanisms such as BRCA reversion and fork protection have motivated combinations including niraparib plus pembrolizumab and olaparib plus durvalumab, both demonstrating promising activity [119,120,121,122]. Furthermore, CADD-guided anti-efflux strategies and target nomination have been applied to resensitize TNBC to conventional agents such as doxorubicin [123].

## 6. CADD Key Technologies and Limitations

Computer-aided drug design (CADD) integrates a broad spectrum of computational techniques that together form the backbone of modern drug discovery. Among these, docking remains a fundamental tool, predicting ligand-target binding poses and affinities and enabling efficient VS of large compound libraries [124,125]. Classical docking programs such as AutoDock are still widely used, while newer deep learning-based frameworks, including DiffDock and EquiBind, have improved docking speed and pose accuracy by leveraging generative and geometric learning strategies [126,127]. Relative to conventional search-and-score procedures, EquiBind directly proposes a one-shot pose using an SE(3)-equivariant geometric model [37], whereas DiffDock treats docking as a generative diffusion process and samples plausible poses together with confidence estimates [32]; in practice, both are typically paired with physics-based rescoring and short MD to reach decision-grade reliability. MD simulations complement docking by capturing conformational flexibility, solvation, and entropic effects that static docking cannot fully address [128,129]. These simulations are particularly valuable in oncology, for example in evaluating the stability of PROTAC-induced ternary complexes where cooperative interactions determine degradation efficacy [130]. Recent advances in enhanced sampling and relative binding free energy (RBFE) methods have further improved the predictive accuracy and scalability of MD-based approaches [129,131]. A concise hybrid workflow is therefore practical: learning-based pose proposal (EquiBind or DiffDock), physics-based rescoring, short MD relaxation, and RBFE on shortlisted complexes [32,37,128,129,130,131]. Typical failure modes include out-of-distribution pockets, protonation or tautomer ambiguity, and induced fit; brief side-chain repacking or short MD can mitigate these issues before free-energy calculations [128,129].

To provide a comparative overview of how these technologies are applied across luminal, HER2^+^, and TNBC, Table 1 summarizes representative strategies, computational methods, and drug examples. For instance, in luminal breast cancer, SERD development has benefited significantly from docking, VS, and MD simulations applied to ESR1 mutations [48,49,50,51,52,53,54,55,56,57,58,59]. In HER2^+^ subtypes, docking and pharmacophore modeling have guided antibody optimization and kinase inhibitor refinement [71,72], while PROTAC design has employed linker optimization and ternary complex modeling [75,76]. For TNBC, CADD methods have supported pathway-focused targeting (e.g., PI3K/AKT, DDR, BRD4), PROTAC design against transcriptional regulators, and integration of radiogenomics for patient stratification [88,89,90,91,92,93,94,95,96,97,98,99,112]. These examples illustrate how each subtype leverages distinct CADD modalities to address unique therapeutic challenges.

**Figure 4:**
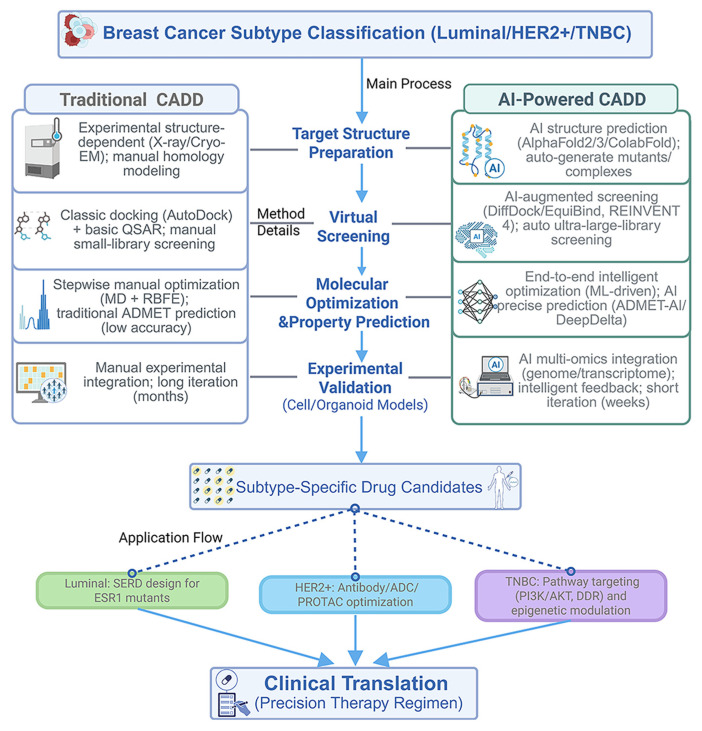
Traditional and AI-powered CADD pipelines for subtype-guided breast cancer drug discovery. Under a breast-cancer subtype framework, both conventional and AI-enabled CADD workflows integrate structure preparation, virtual screening, molecular optimization and property prediction, and experimental validation, converging on subtype-specific drug candidates that inform clinical translation. Created in BioRender. Yang, J. (2025) https://BioRender.com/09rr139.

To complement Table 1, Figure 4 contrasts traditional, AI-driven, and hybrid CADD workflows at a glance. Traditional CADD, centered on docking and MD simulations, established the foundation for design against established breast-cancer targets [124,125,128,129,130,131]. Its reliance on handcrafted features and iterative sampling can limit performance for subtype-specific and highly flexible targets and for ultra-large libraries. AI-enabled CADD addresses these constraints through generative and geometric learning that capture complex spatial patterns in protein-ligand interactions and that can integrate multi-modal inputs, including imaging and digital pathology, to support end-to-end optimization from subtype identification to compound design [83,84,132,133,134]. In translational settings, these capabilities provide practical routes to long-standing challenges: in luminal disease, strategies contextualized to ESR1-mutation resistance, including SERD design and prioritization [48,49,50,51,52,53,54,55,56,57,58,59]; in HER2-positive disease, response prediction and patient stratification aided by quantitative pathology, including TIL assessment [78,79]; and in TNBC, de novo generation and prioritization of candidates for difficult pathways and targets considered undruggable [88,89,112,132,133,134]. Traditional and AI-driven approaches are complementary: interpretable, physics-based models provide mechanistic checks, whereas AI modules accelerate pose generation, library triage, and property optimization, together supporting subtype-guided precision therapy.

AI and ML have been increasingly incorporated into multiple stages of CADD. High-throughput prediction of ADMET has been enabled by platforms such as ADMET-AI, although variations in predictive accuracy across endpoints have been reported [126,135]. For very large libraries, Deep Docking enables iterative, model-guided triage to prioritize compounds at scale and reduce explicit docking load [136]. For property prediction and lead optimization, Chemprop employs directed message-passing neural networks and integrates with QSAR and ADMET workflows [137]. Molecular property differences between related compounds have been captured by tools such as DeepDelta, and fine-grained optimization during lead development has been supported. Beyond ADMET profiling, ML has also been employed for drug repurposing, de novo molecule generation, and multi-parameter optimization, thereby providing systematic decision support across the discovery pipeline [132,133,134]. QSAR modeling continues to serve as a cornerstone of ligand-based design, and recent advances in deep QSAR have been achieved through the use of large datasets and neural networks [138]. In addition, large-scale docking has been accelerated by AI-powered engines such as CarsiDock, in which pretraining on millions of protein-ligand complexes has been utilized to improve speed and generalizability [133].

AI models have also been applied to resistance prediction, in line with the applications summarized in Table 1. Classical algorithms such as random forests and support vector machines (SVMs) have been used to integrate molecular and omics data, and resistant versus sensitive subgroups have been stratified accordingly [15]. More recently, graph neural networks (GNNs) have been implemented to enhance representation learning for complex molecular graphs, and superior predictive performance in resistance-associated tasks has been reported [8]. Through these approaches, therapeutic resistance has been anticipated computationally, and rational strategies for drug design and patient selection have been informed.

**Figure 5:**
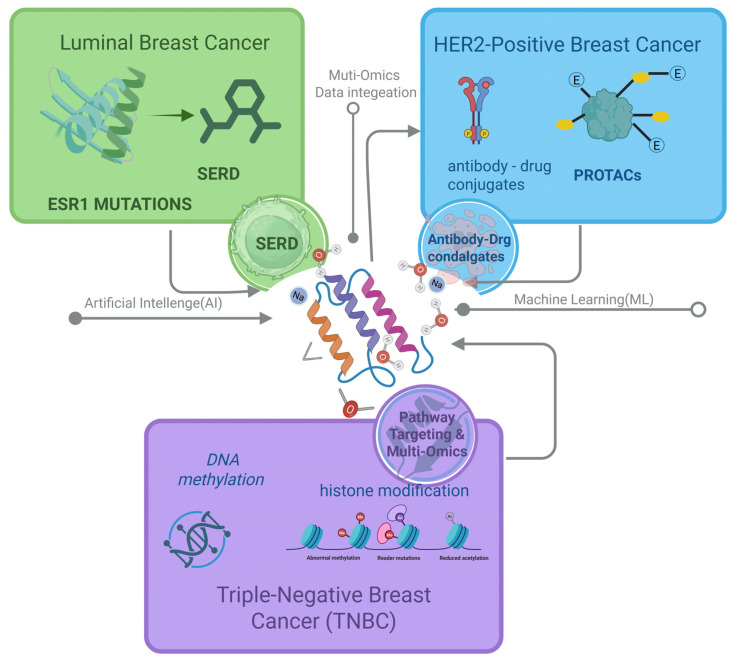
Subtype-guided CADD map for breast cancer. This schematic integrates AI, ML, and multi-omics data to guide CADD across breast cancer subtypes. In luminal, ESR1 mutations that drive endocrine resistance are addressed with SERDs targeting the ER. In HER2^+^, structure-guided design and optimization prioritize ADCs and PROTACs. In TNBC, pathway-focused strategies emphasize epigenetic modulation of DNA methylation and histone modifications to enable rational combinations and patient stratification. Created in BioRender. Yang, J. (2025) https://BioRender.com/lyuxbcp.

Recent advances extend beyond pose generation. AlphaFold 3 expands structural prediction to protein-DNA/RNA/ligand assemblies, enabling more reliable docking inputs [126,139,140]. Similarly, open-source REINVENT 4 standardizes reinforcement learning pipelines for generative molecular design, facilitating robust production-level applications [131,139,140]. Two-stage frameworks such as DeltaDock integrate pocket prediction with docking, significantly improving blind docking accuracy [131,139,140]. Moreover, ML-guided ultra-large VS with conformal prediction has enabled triaging of multi-billion compound libraries with markedly reduced computational cost while maintaining high sensitivity [119,139,140]. Thermodynamics-focused methods have also matured: automated workflows like PyAuto FEP facilitate RBFE calculations [117,139,140], while next-generation force fields such as OpenFF 2.0 (Sage) improve ΔΔG accuracy in protein-ligand systems [117,139,140]. Figure 2 presents an integrated workflow that illustrates how docking, MD, QSAR, and AI-based methods synergistically accelerate subtype-specific drug discovery. Together, these computational technologies support systematic and iterative cycles of VS, lead optimization, and candidate prioritization, thereby expediting the development of effective therapies for breast cancer. Figure 5 provides a subtype-guided CADD map that integrates AI/ML and multi-omics inputs, linking luminal ESR1-mutant SERD design, HER2-positive ADCs and HER2-directed PROTACs, and TNBC pathway- and epigenetics-oriented strategies.

Despite its transformative potential, CADD still faces critical limitations that constrain its predictive reliability and translational impact. A primary bottleneck is its dependence on structural data quality. Predictions rely heavily on high-resolution target structures, yet many membrane proteins, intrinsically disordered proteins, and multi-subunit complexes remain poorly resolved. While cryo-EM has significantly improved coverage of druggable targets [124], unresolved loops or flexible domains can introduce uncertainty into docking and simulation outcomes. Recent evaluations of AlphaFold-Multimer and AlphaFold 3 suggest that while predicted models provide valuable starting points, their suitability for docking and dynamics varies by target class and requires cautious validation [125,126].

Another major limitation lies in toxicity and safety prediction. Although ML-driven ADMET models such as ADMET-AI have improved throughput, their prospective accuracy remains uneven across pharmacological endpoints [126,135]. For example, predictions for solubility and lipophilicity are relatively reliable, but those for hepatotoxicity or cardiotoxicity often lack sufficient accuracy, necessitating experimental confirmation. This gap prolongs the transition from computational hits to viable drug candidates [126].

CADD also struggles with multi-target and systems-level design. Traditional tools are best suited for single target-ligand interactions, yet cancer biology is inherently multi-dimensional, involving crosstalk between signaling pathways, protein–protein interactions, and microenvironmental factors. For example, TNBC drug discovery increasingly requires integration of PARP inhibitors with modulators of immune checkpoints or epigenetic regulators, which current pipelines only partially capture. Network pharmacology and systems pharmacology approaches have been proposed [130], but these remain in early stages and are computationally intensive.

Finally, algorithmic bias and lack of generalizability remain significant hurdles. Deep learning-based docking and scoring functions have shown promising results in benchmarks but often fail to generalize to novel targets or chemical scaffolds [127,131]. Dataset bias, insufficient chemical diversity, and lack of transparency can all contribute to misleading predictions. Reviews of deep QSAR and AI in drug discovery emphasize the need for better data curation, robust benchmarking, and uncertainty quantification to ensure reproducibility [134,136,137,138]. To improve generalizability and interpretability, explicit uncertainty quantification (e.g., conformal prediction, ensembles) and prospective validation on out-of-distribution targets are recommended. Adoption of curated, harmonized datasets will be critical to reduce bias and improve reproducibility. When relying on predicted structures (e.g., AlphaFold-Multimer/3) for docking or MD, confidence annotation and targeted relaxation remain essential, as predictive accuracy varies by target class [32,139,140,141,142,143,144]. Addressing these limitations will be critical for translating computational predictions into clinically relevant outcomes.

## 7. Conclusions and Future Perspectives

CADD has been increasingly recognized as a transformative framework in breast cancer therapeutics, enabling rational, efficient, and subtype-specific strategies. In luminal disease, the development of next-generation SERDs has been advanced into clinical practice. In HER2^+^ subtypes, antibody engineering and PROTAC-based degraders have been refined through structure-based modeling. In TNBC, the discovery of epigenetic modulators, pathway-specific inhibitors, and multi-omics-driven stratification approaches has been facilitated by CADD. Collectively, these achievements demonstrate how molecular insights have been systematically translated into therapeutic opportunities through computational approaches. Building on these foundations, learning-based modules now complement classical pipelines: geometry-aware pose generation (EquiBind) and diffusion-based docking (DiffDock), ultra-large-library triage (Deep Docking), and graph-network property prediction (Chemprop) can serve as fast front-ends to physics-based refinement [32,37,124,125,126,127,128,129,130,131,132,133,134,135,136,137,138]. Together with the workflow and subtype overviews in Figure 4 and Figure 5, these developments illustrate how computational methods translate molecular insight into therapeutic opportunities.

Nevertheless, significant challenges remain. The predictive reliability of computational pipelines is still constrained by incomplete structural coverage, and unresolved regions of key drug targets continue to limit accuracy [124,126,127]. The difficulties associated with toxicity prediction, multi-target design, and algorithmic bias also persist, thereby restricting clinical translation [66,134]. Resistance mechanisms, particularly in TNBC and HER2^+^ cancers, have further underscored the complexity of tumor evolution, highlighting the necessity for CADD strategies that incorporate systems-level modeling and patient heterogeneity [128]. In addition, while AlphaFold 3 has provided a breakthrough in structural prediction, debates have been reported regarding its accuracy in predicting complex assemblies [126,139,140], emphasizing that experimental validation remains indispensable. When predicted structures are used, confidence annotation, targeted relaxation, and short MD are needed to qualify models for docking or RBFE calculations, with explicit handling of uncertainty to avoid over-interpretation [128,129,130,131,139,140].

Near-term progress is expected from tighter integration of multi-omics data, digital pathology, and patient-derived models with hybrid AI-plus-physics workflows. In practice, pose proposals from EquiBind or DiffDock, library triage by Deep Docking, and Chemprop-style property models can be coupled to rescoring, short MD, and RBFE to improve decision quality and efficiency [32,37,128,129,130,131,132,133,134,135,136,137]. Explainable and federated learning, together with uncertainty quantification, are likely to be piloted for resistance prediction and patient selection in the next few years [8,137]. In parallel, organoid-based experimental platforms are expected to close the loop with computation and increase translational fidelity.

From an economic perspective, the potential of CADD to reduce costs in early-stage drug discovery has been highlighted, particularly by lowering attrition rates and decreasing reliance on extensive wet-lab screening [136]. Real-world implementation will, however, require standardized frameworks for multi-omics integration, regulatory approval pathways for AI-designed candidates, and scalable infrastructure that bridges computation with experimental validation. Market analyses have projected sustained double-digit growth for AI-powered CADD platforms [134], but long-term success is likely to depend on transparent validation standards and the establishment of trust across the drug discovery ecosystem. Overall, converging technologies, as summarized in Figure 4 and Figure 5, are poised to benefit subtype-aware pipelines in luminal, HER2^+^, and TNBC settings and to accelerate the development of more precise and durable therapies.

## Figures and Tables

**Table 1:** Comprehensive summary of CADD-driven strategies in different breast cancer subtypes.

Subtype and Features	Design Focus	CADD Methods	Tools/Software/Servers	Representative Agents
Luminal-ER^+^/PR^+^; typically HER2^−^; frequent ESR1 and PIK3CA mutations; endocrine resistance/brain exposure considerations	ER Degradation	Docking	AutoDock Vina; Glide; GOLD; GNINA	Elacestrant [48,49,50,51,52]; GDC-0810 [53]; Camizestrant [54]; Imlunestrant [55]; AZD9496 [56,57,58,59,60,61,62,63,64]
		VS	ZINC; Enamine REAL	Elacestrant [48,49,50,51,52]; Imlunestrant [55]
		MD simulations	GROMACS; AMBER; OpenMM	Elacestrant [48,49,50,51,52]; Imlunestrant [55]; ESR1-Y537S/D538G mutants [58,59]
	Structural Insights & Rational Design	Cryo-EM	RELION; cryoSPARC; EMDB	GDC-0810 structural insights [57]
		X-ray Crystallography	Phenix; Coot; PDB	GDC-0810 [53]; ESR1 mutant scaffolds [58,59]
		NMR	TopSpin; NMRPipe	Allosteric SERD scaffolds [52]
		AlphaFold Predictions	AlphaFold/ColabFold; MODELLER; SWISS-MODEL; Rosetta	ESR1-Y537S/D538G mutant studies [58,59]
		Free-Energy Calculations	FEP+; PyAutoFEP (OpenMM)	GDC-0810 [53]; conformational refinements [57,58]
	Pharmacokinetics/ADME Optimization	QSAR Modeling; ADME PredictionML Models	scikit-learn; Chemprop; DeepChem; pkCSM; ADMETlab; ADMET-AI	Elacestrant [48,49,50,51,52]; AZD9496 [60,61,62,63,64]
		PBPK simulations	GastroPlus; Simcyp	Elacestrant [48,49,50,51,52]
HER2^+^-HER2 overexpression/amplification (IHC 3^+^ or ISH^+^); frequent PIK3CA co-alterations; spatial heterogeneity; higher CNS risk^+^	Antibodies, ADCs, and TKIs	Docking	AutoDock Vina; Glide; GNINA	Tucatinib [72]; Lapatinib [72]; Neratinib [72]
		Pharmacophore Modeling	Rosetta; MODELLER; SWISS-MODE	Trastuzumab [72]; Pertuzumab [72]
		MD	GROMACS; OpenMM	T-DM1 [72]; T-DXd [78]
		AI-Assisted Antibody Design	RosettaAntibody; DeepAb; IgFold	Zanidatamab [132]
	Targeted HER2 Degradation (PROTACs)	Linker Optimization	OpenMM; Rosetta	Tucatinib-based PROTAC [69]
		E3 Ligase Selection	PRosettaC; Rosetta	HER2-directed PROTACs [75,76]
		Ternary Complex Docking & MD	Rosetta; OpenMM	HER2 PROTAC prototypes [75,76]
	Multi-Omics Integration	Multi-Omics ML	scikit-learn (survival/classifiers)	PI3K-mutant subgroup predictions [77]
		AI-Driven Pathology	QuPath; HALO AI; MONAI; CLAM	Trastuzumab deruxtecan-sensitive subtypes [78]
		Digital Pathology (HER2 IHC)	QuPath; MONAI	Automated HER2 IHC scoring [65]
TNBC-ER^−^/PR^−^/HER2^−^; often basal-like; BRCA1/2 or high HRD; genomic instability; TROP-2 expression	Pathway Targeting (PI3K/AKT, DDR, Epigenetic)	Docking	AutoDock Vina; Glide	AKT inhibitors [73,75]
		MD	OpenMM; GROMACS	PARP inhibitors [85,86]; PARP/HDAC hybrids [79,83]
		ADME/T Profiling	pkCSM; ADMETlab; ADMET-AI	BET inhibitors [93,94,96]
		Dual-Warhead Modeling	Rosetta; OpenMM	EGFR/BRD4 dual inhibitors [84,86]; JAK2/BRD4 dual inhibitors [95]
	PROTACs for Transcriptional Regulators	Linker Design	OpenMM; Rosetta	BRD4 PROTACs (CRBN recruiting) [75,76]
		Ternary Complex MD	OpenMM; Rosetta	BRD4 PROTACs suppressing KLF5 [75,76]
		Solvent Mapping	FTMap; MixMD	BRD4 PROTACs in TNBC models [75,76]
	Multi-Omics Integration	Subtype-Guided Docking	AutoDock Vina; Glide	BRD4 inhibitors in basal-like TNBC [95]
		ML Predictors	Chemprop; scikit-learn	Subtype-specific stratification [95]
		Radiomics/Radiogenomics	PyRadiomics; scikit-image; MONAI	Imaging-guided therapeutic ranking [95]

Abbreviations: estrogen receptor (ER); selective estrogen receptor degrader (SERD); human epidermal growth factor receptor 2 (HER2); triple-negative breast cancer (TNBC); antibody-drug conjugate (ADC); tyrosine kinase inhibitor (TKI); proteolysis-targeting chimera (PROTAC); molecular dynamics (MD); free-energy perturbation (FEP); absorption, distribution, metabolism, excretion, and toxicity (ADMET); physiologically based pharmacokinetics (PBPK); DNA damage response (DDR); bromodomain-containing protein 4 (BRD4); poly(ADP-ribose) polymerase (PARP); epidermal growth factor receptor (EGFR); Janus kinase 2 (JAK2); homologous-recombination deficiency (HRD); central nervous system (CNS); immunohistochemistry (IHC); in situ hybridization (ISH); protein data bank (PDB); electron microscopy data bank (EMDB).

## Data Availability

No new data were created in this study. Data sharing is not applicable to this article.
